# An Analysis of Entropy-Based Eye Movement Events Detection

**DOI:** 10.3390/e21020107

**Published:** 2019-01-24

**Authors:** Katarzyna Harezlak, Dariusz R. Augustyn, Pawel Kasprowski

**Affiliations:** Institute of Informatics, Silesian University of Technology, Akademicka 16, 44-100 Gliwice, Poland

**Keywords:** eye movement, eye movement events detection, approximate entropy, multiresolution analysis, time-scale decomposition

## Abstract

Analysis of eye movement has attracted a lot of attention recently in terms of exploring areas of people’s interest, cognitive ability, and skills. The basis for eye movement usage in these applications is the detection of its main components—namely, fixations and saccades, which facilitate understanding of the spatiotemporal processing of a visual scene. In the presented research, a novel approach for the detection of eye movement events is proposed, based on the concept of approximate entropy. By using the multiresolution time-domain scheme, a structure entitled the Multilevel Entropy Map was developed for this purpose. The dataset was collected during an experiment utilizing the “jumping point” paradigm. Eye positions were registered with a 1000 Hz sampling rate. For event detection, the *knn* classifier was applied. The best classification efficiency in recognizing the saccadic period ranged from 83% to 94%, depending on the sample size used. These promising outcomes suggest that the proposed solution may be used as a potential method for describing eye movement dynamics.

## 1. Introduction

The analysis of eye movement has attracted a lot of attention recently in exploring areas of people’s interest, cognitive ability, and skills. Growing interest in this field has been triggered by the development of technological solutions, as well as methods for eye-movement processing. This has resulted in the decreasing cost of eye trackers–devices which enable eye-movement registration. As a consequence, substantially more institutions have obtained a new tool for dealing with their quotidian tasks. For example, eye-movement technology may be used for things such as ascertaining the usability of various interface types [1,2], recognizing visual patterns when reading [3,4] or searching for interesting information, and differentiating between experts and novices [5,6]. Usage of eye-tracking solutions also plays an important role in medicine [7], psychology [8], and cognitive studies [9]. This growing interest in eye-tracking use drives, in turn, further advances for new solutions.

### 1.1. Eye Movement Processing

**Figure 1:**
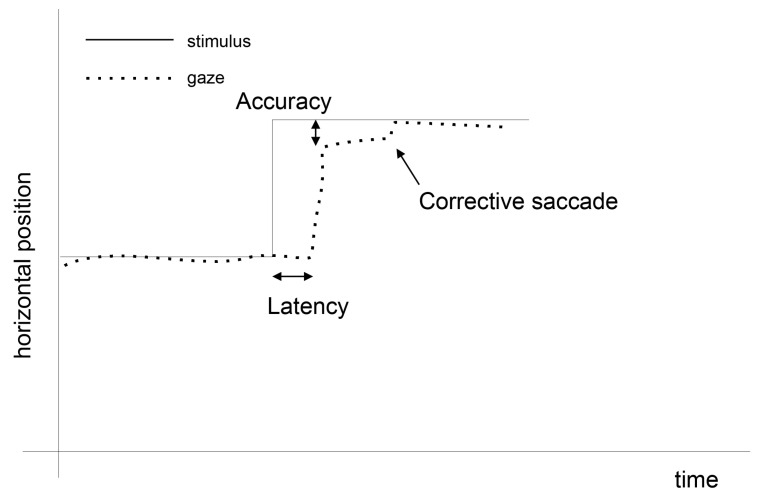
An example of eye movement events. At the beginning the eye is focused on the stimulus. When the stimulus moves to another location, the eye moves as well, yet after a period of saccadic latency.

The basis for eye-movement usage in the aforementioned applications is detection of its main components: fixations and saccades. A fixation is identified when the eye is almost motionless and acquires information from an observed scene. A saccade is a quick movement between fixations, during which no information is taken [10]. Moreover, due to the construction of the eye and its way of functioning, within each fixation, additional micro-movements are present: tremors, microsaccades, and drifts [11,12], the discovery of which requires high-frequency eye trackers. Sometimes eye motions are imposed by a stimulus movement, and the user has to fixate on the place where the stimulus appears. As the brain needs some time to react to this change, a phenomenon called *saccadic latency* occurs. It is defined as the time from a stimulus presentation to the commencement of the saccade [13]. This occurrence, together with the preceding fixation and the following saccade, is presented in Figure 1.

The ability to select fixations and saccades facilitates understanding of the spatiotemporal processing of a visual scene. Several methods have been developed for this purpose, a review of which is presented in [14]. There are methods, based on various user-defined thresholds, among which the most well-known are Dispersion Threshold Identification (I-DT) or Velocity Threshold Identification (I-VT). They take advantage of the fact that fixation movements are of low velocities, with points placed within a small area, while saccades are characterized by high velocities and a higher dispersion of points [15]. One of the major drawbacks of these solutions is the dependency of the obtained results on user-defined thresholds and the lack of their commonly-accepted values. As a result, various ranges in these parameter values may lead to a diversity in results—namely, the number and duration of fixations and saccades. This problem has been alleviated by the application of adaptive thresholds, which was investigated, for example, in [16,17]. However, these approaches were judged as having some limitations in a chosen range of noise levels. Moreover, some solutions for the detection of smooth pursuit and post-saccadic oscillations [18,19] and fixations in the scope of infant research [20] were developed, but were dedicated to a particular application.

Regardless of the many proposed approaches, no standard methods have been established yet. Hence, the authors in [14] decided to introduce a new technique based on machine learning. They utilized a 14-dimensional feature vector, including, for example, such measurements as: eye-movement sampling rate, samples dispersion, standard deviation, and various types of distances between samples in regard to a defined window size. They concluded that machine-learning techniques led to superior detection compared to the current state-of-the-art events detection algorithms, and that those techniques could even reach the performance of manual coding. This was the motivating factor in undertaking the presented studies, aimed at checking the possibility of utilizing machine learning for recognizing eye movement events based on their dynamics.

Recently, eye movement has been explored in this field with the usage of the nonlinear time series analysis. In such an approach, gaze data are perceived as representatives of biological nonlinear signals. The studies conducted in this field are aimed at ascertaining the dynamic characteristics of eye movement. For this purpose, gaze locations [21,22] and eye movement velocities during fixation [23,24] were inspected in terms of the existence of chaotic features. One of the indicators of such a system’s nature is the Largest Lyapunov Exponent (LLE) when assuming positive values [25]. Analysis of this indicator in all the aforementioned studies demonstrated chaos in the parts of eye movement under consideration. Additionally, in [26], fractal dimension and sample entropy were evaluated for eye movement velocity during fixations. These factors, as well as the LLE, were examined, taking different eye movement signal segments into account. Their analysis showed that various eye movement parts represented different characteristics. This fact has become the predominant motivating factor for further exploration of dynamic eye movement features. Approximate entropy was chosen as the most suitable [27]. This choice was motivated by the literature review. We searched for a method which allowed for calculation entropy for relatively short data series, and which would enable us to trace changes in entropy values with a system evolution. Thus, based on the analysis of previous applications of approximate entropy in the exploration of biological signal features, it was assumed to be an appropriate solution. The group of research utilizing this method will be described in the following section.

### 1.2. Approximate Entropy Applied for Biological Signals

Approximate entropy has proved useful in examining dynamic features in various biological signals. For example, it was calculated in order to determine the effects of respiratory failure on respiratory rate patterns and tidal volume patterns [28]. Bruhn et al. [29] applied this method to assess the electroencephalogram (EEG) during higher anaesthetic concentrations. They discovered an increase in EEG regularity with increasing drug concentrations. Promising results were also obtained with the usage of the automated epileptic EEG detection system that utilizes approximate entropy [30]. In [31], the relationship between approximate entropy and standard deviation of a gait variable for young and elderly gait patterns was investigated. The authors claimed that the obtained results may be potentially useful for the early diagnosis of common gait pathologies. Approximate entropy was also utilized in order to inspect ECG (Electrocardiography) signals in the supine and the sitting position [32]. These two conditions were selected according to the expectation that they would be characterized by different levels of entropy. The studies confirmed these expectations; however, the type of changes depended on the parameters used. Furthermore, the evaluation criteria of cognitive workload based on the approximate entropy was proposed in [33]. They were meant to be applied in the estimation of ergonomics in human-interface interactions.

### 1.3. Contribution

Within the group of research concerning exploration of biological signals with the usage of approximate entropy, we have not found any pertaining to eye movement. The idea introduced in this paper is to apply this method for detecting eye-movement signal events. According to the authors’ knowledge, such an approach has not yet been investigated. Thus, the contribution of these studies may be defined as follows:

- Application of approximate entropy for building a general description of eye movement dynamics;
- Introducing a multilevel entropy map representing an eye movement characteristic obtained between successive stimulus appearances;
- Introducing a method utilizing the approximate entropy map, which may prove useful in determining saccade periods in eye movement.

## 2. Materials and Methods

### 2.1. Description of the Experiment

**Figure 2:**
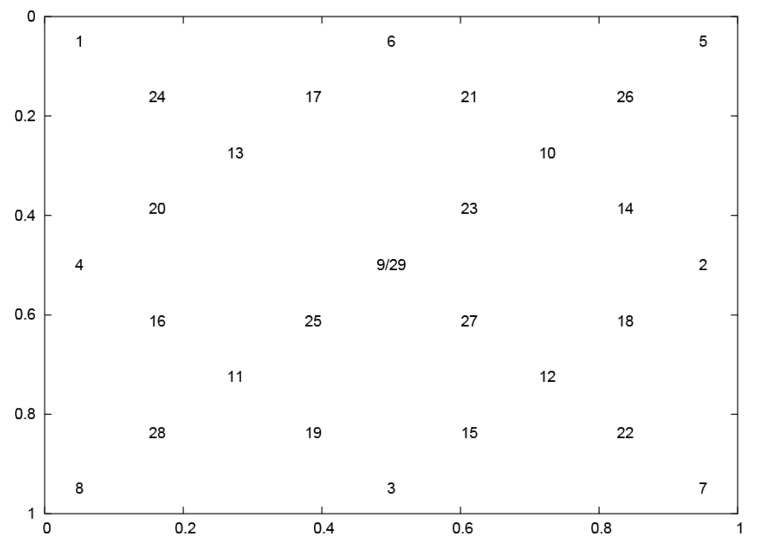
Layout of stimuli applied during experiments, based on the universal scale. The top-left corner of the screen is represented by coordinates (0.0, 0.0), and the bottom-right corner by (1.0, 1.0), respectively.

The eye movement dataset—used in the presented studies (see Supplementary Materials)—was collected during an experiment in which the participants were asked to look at a point jumping between 29 positions ($N_{pp}$) on the screen, as shown in Figure 2.

**Figure 3:**
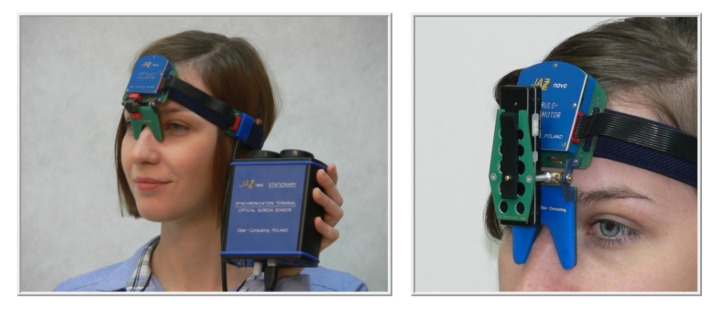
Head-mounted JAZZ -novo eye tracker [34] used in the research.

The point was displayed in each location for 3 s. Eye positions were registered by means of the head-mounted JAZZ-novo eye tracker [34], able to register eye movements with a sampling rate equal to 1000 Hz (Figure 3).

24 participants ($N_{p}$) aged between 22 and 24 with normal vision were engaged in the experiment, which consisted of two sessions, three weeks apart ($N_{spp}$). As a result, $N_{ps}$ participants’ sessions were gathered, $N_{ps}=N_{p}*N_{spp}$, each of which comprised subsequent eye positions for all points’ locations. Such a dataset was divided into $N_{EMs}$ eye movement series ($N_{EMs}=N_{p}*N_{spp}*N_{pp}$), which are later referred to as *EM series*. Each of them included one participant’s eye positions registered between the moment of the appearance of the stimulus and the time of its position change. The inspection of the collected signals revealed, in the case of two eye movement (EM) series, there were some problems regarding the recording procedure; therefore, they were removed from further analysis. Thus, the $N_{ps}$ value decreased by 2, and $N_{EMs}$ by $2\times N_{pp}$.

Subsequently, by applying the standard procedure of the two-point signal differentiation, the first derivative of horizontal coordinates for each EM series was obtained. Thus, each series corresponds to the velocity of eye movement in a period between two appearances of the stimulus. This quantity was used to avoid over-learning; defined as too much adaptation to the specific map of point locations, presented in Figure 2. It renders the method more general in terms of the independence of stimulus positions.

### 2.2. The Method

**Figure 4:**
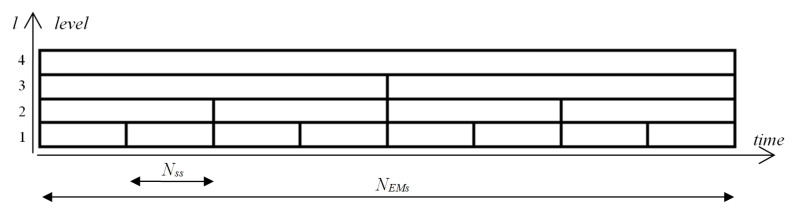
Segments in the multilevel time-scale structure.

The aim of this study, as previously mentioned, was to provide a description of some general features of eye movement dynamics, and to develop mechanisms for utilizing them in detecting some characteristic elements in eye-movement signals. For this reason, a multiresolution representation of entropy was defined. It may be expected that differences in the dynamic of a signal course may impact entropy values calculated for different time segments (frames). This led us to form a concept of a time-scale decomposition of the entropy where the signal at the (l+1)-th level of the scale would be divided into two shorter segments at the *l*-th level. The smallest size of segments is denoted by $N_{ss}$. Such segments are used at the first level of the scale. It determines that there is $N_{EMr}/N_{ss}$ number of segments at the first level of the scale (see Figure 4). The size of the segment at the *l*-th level equals $2^{l-1}\times N_{ss}$.

**Figure 5:**
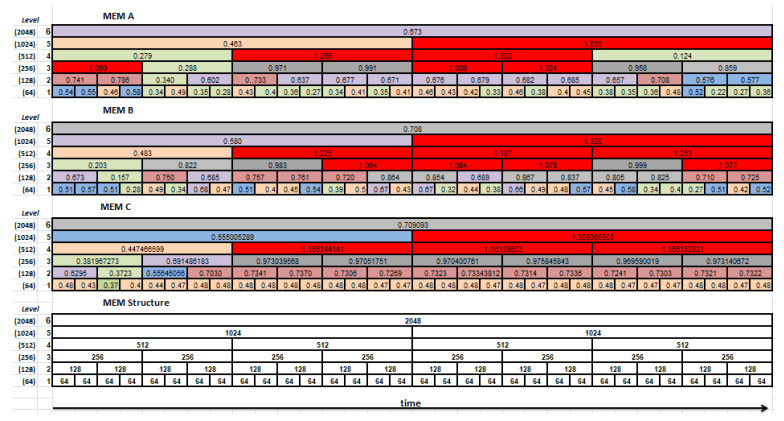
The multilevel entropy maps obtained for the two chosen sets (maps MEM A and MEM B) and averaged over all eye movement (EM) series (map MEM C). The table at the bottom presents the maps’ structure.

The entropy is computed separately for each segment at all levels of the scale. This resulting structure is called a *Multilevel Entropy Map* (MEM), examples of which are shown in Figure 5. The above-described technique of multiresolution analysis, where a time-domain and scale-domain are used, is well-known, e.g., in the 1-D wavelet decomposition of signals.

For each cell of MEM, approximate entropy was calculated. This method was proposed in [27] as a measure of regularity to quantify levels of complexity within a time-series. For a given integer parameter *m* and a positive real one *r*, for a time-series u(1),…,u(N) in **R**, the sequence of vectors x(1),…,x(N−m+1) in **R**${}^{m}$ can be defined, where x(i)=[u(i),…,u(i+m−1)].

For 1≤i≤N−m+1, using:

(1) $$C_{i}^{m}\left(r\right)=\frac{\mathrm{number}\;\mathrm{of}\;j:d[x(i),x(j)]\leq r}{N-m+1}$$

where the distance *d* is defined as follows:

(2) $$d\left[x\left(i\right),x\left(j\right)\right]=\max_{k=1,2,\ldots ,m}(|u\left(i+k-1\right)-u\left(j+k-1\right)|)$$

and

(3) $$\Phi^{m}\left(r\right)={\left(N-m+1\right)}^{-1}\sum_{i=1}^{N-m+1}\log C_{i}^{m}\left(r\right)$$

the approximate entropy can be obtained:

(4) $$\mathrm{ApEn}\left(m,r\right)=\lim_{N\to \infty }\left[\Phi^{m}\left(r\right)-\Phi^{m+1}\left(r\right)\right].$$

The asymptotic formula in Equation (4) cannot be used directly, and the estimator of approximate entropy (for sufficiently large values of *N*) is defined as:

(5) $$\mathrm{ApEn}\left(m,r,N\right)=\Phi^{m}\left(r\right)-\Phi^{m+1}\left(r\right).$$

There is usually significant variation in ApEn(m,r,N) over the range of *m* and *r*, when a particular system is taken into consideration [35]. Thus, for approximate entropy comparisons, *m* and *r* should be fixed. In the studies described in [36,37] it was shown that choosing: a value from {1,2} for parameter *m*, and the standard deviation of the entire time series multiplied by a value from range 0.1 and 0.25, for parameter *r*, ensures good statistical validity of ApEn(m,r,N) from Equation (5).

Additionally, for entropy comparisons, it is suggested to have the number of samples (*N*) fixed [36], wherein datasets with lengths between 75 to 5000 data points may be used [36]. A review of the previously described research revealed that in all of the presented studies, the first parameter *m* was set to 2, as suggested for biological signals [27]. Hence, we decided to apply the same value for the purposes of this research. In the case of the second parameter *r*, approximate entropy was evaluated for three multipliers of dataset standard deviation: {0.1, 0.15, 0.2}. The obtained results were similar, both in values and characteristics; therefore, the presentation of all of them was not justified. Based on the recommendation to apply such a multiplier, which maximizes approximate entropy [32], the value 0.2 was used in further analysis.

The number of eye-movement recordings ($N_{EMr}$) for building the entropy map was set at 2048. This size was calculated by taking into account: (1) the length of EM series, (2) the frequency of sampling fs = 1000 Hz, and (3) aligning to the nearest—and not less—multiple of powers of two. The structure assumed for MEM, and the number of its levels, influenced segment size on each level of the scale. *N* (in Equation (1)) equals $N_{ss}\times 2^{\left(l-1\right)}$ for l=1…6, i.e., N=64,…,2048. Taking the number of recordings used in the research into consideration, the $N_{ss}$ on the 1th level equals 64. The use of such a small *N* at the 1th level results from the intention of obtaining the highest possible resolution of MEM in the time-domain, in accordance with the assumed “powers of two” rule, as well as having a value close to 75, as mentioned above.

The calculations were conducted in the R project with the usage of the *approx_entropy* method available in the *pracma* package [38].

## 3. Results

During the experiments, the multilevel entropy maps were estimated for each EM series separately and subsequently averaged over all $N_{EMs}$ EM series, for each level and its segments independently. The obtained results for two chosen sets (MEM A, MEM B) and the averaged one (MEM C) are presented in the form of the entropy maps in Figure 5. Cells of the maps are colored according to the values of the estimated entropy:

- Green was used when the entropy was lower than 0.4;
- Brown was dedicated to entropy values between 0.4 and 0.5;
- [0.5,0.6) was highlighted by blue;
- [0.6,0.7) by light violet;
- [0.7,0.8) by light burgundy;
- [0.8,0.9) and [0.9,1.0) by light and dark gray, respectively;
- Values greater than 1 by red.

**Figure 6:**
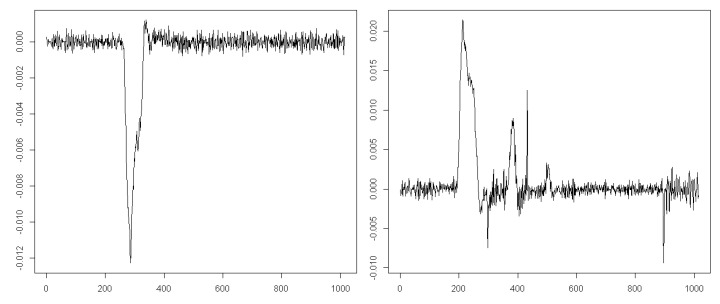
Two example courses of eye-movement velocity with the saccadic period. The horizontal axis represents time, and the vertical one represents velocity of eye movement.

A comparison of the first two entropy maps prepared for two example datasets with their velocity signals (Figure 6) allows putting forward the hypothesis that it is feasible to recognize the saccade period based on the approximate entropy estimated for this signal. During the eye movement presented on the left-hand side in Figure 6, the saccade is invoked later than in the other case, visible on the right-hand side. This difference seems to be reflected in the entropy maps of these signals. It is represented by lower entropy values marked by green in the first, second, and third levels of the map.

**Figure 7:**
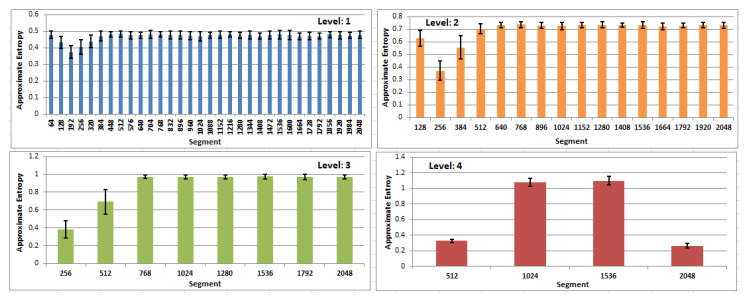
Bars showing the averaged values of approximate entropy for four levels of the entropy map—64, 128, 256, 512—and for all segments within each level, with whiskers representing standard deviations.

Additionally, the averaged entropy values with their standard deviation, for the first four levels of MEM and for all segments, are presented in Figure 7. In all charts, lower entropy values may be observed in the beginning segments of eye movements. Small values of standard deviation, relative to the values of averages, allow the expectation that values in multilevel entropy maps describe an eye-movement well and in general (regardless of a specific user and a particular EM series). This, in turn, allows the anticipation of some ability to detect a saccadic period.

In order to check the significance of the achieved differences, statistical tests were conducted. The analysis was performed with the usage of the ANOVA test, although the Shapiro test did not confirm the results’ normality. However, exploration of their density plots justifies such a method selection. Detailed, between-group dependency was explored with Tukey’s HSD (Tukey’s Honest Significant Difference) test. The tests were conducted under the null hypothesis stating that the means of entropy for segments belonging to one MEM level are the same. A 95% confidence interval was chosen. The outcomes of this analysis revealed that in the case of a segment size equaling to:

- 64, the majority of differences were not significant;
- 128, all differences concerning the first four segments (1–128, 129–256, 257–384, 384–512) turned out to be significant, on the contrary to the group of remaining segments;
- 256, it was similar to set128, where only for the first two segments were significant differences yielded;
- 512, differences were significant only when the first segment was considered;
- 1024, significant differences were disclosed.

### Eye-Movement Events Detection

The differences revealed that in particular segments of eye movements—visible especially at the first 500 ms—there was a motivating factor in undertaking a subsequent step towards the verification as to whether it was possible to recognize the eye movement partly based on its approximate entropy value. For this purpose, the *knn* classifier—for various sets of feature vectors and different values for the parameter *k*—was applied. The values 3, 7, 15, 31, 63, 127, and 255 were chosen for the *k* parameter. The choice of the classifier was based on previously conducted research, in which this method proved useful in eye-movement processing [39].

Several sets of features were defined. Each of them consisted of differently combined MEM segment sizes, belonging either to one or some selected levels of MEM. Set names reflect the used segment sizes: set64, set128, set256, set512, set64_128, set64_128_256, set64_128_256_512, set128_256, set128_256_512, set256_512.

As a result, 10 feature sets were prepared. The sets are:

- Set64, set128, set256, and set512 consisted of only one feature;
- Set64_128, set128_256 and set256_512 consisted of two features;
- Set64_128_256 and set128_256_512 consisted of three features;
- Set64_128_256_512 consisted of four features.

**Figure 8:**
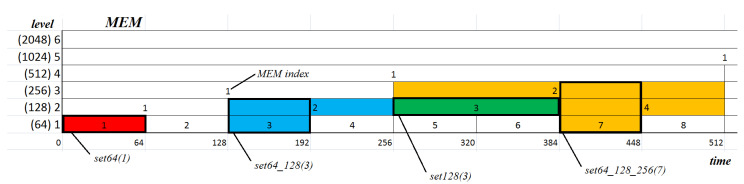
Sample one-, two-, and three-dimensional vectors of features embedded in the MEM structure.

In order to illustrate the approach, some example feature sets have been described in detail:

- The dataset denoted by set64 is based on:
  Only one level of MEM denoted by 64, where the size of segment equals 64, the number of segments equals $N_{EMr}/N_{ss}=32$, a single element of the dataset is a scalar value—e.g., the 1st element is MEM(1,1) (red box in Figure 8);
- The dataset denoted by set128 is based on:
  Only one level of MEM denoted by 128, where the size of segment equals 128, the number of segments equals $N_{EMr}/\left(2\times N_{ss}\right)=16$, a single element of the dataset is a scalar value—e.g., the 3rd element is MEM(2,3) (green box in Figure 8);
- The dataset denoted by set64_128 is based on:
  Two levels of MEM denoted by 64 and 128, where the size of segment equals min(64,128)=64, the number of segments equals $N_{EMr}/N_{ss}=32$, and a single element is a two-dimensional vector of features—e.g., the 3rd element is [MEM(1,3),MEM(2,2)] (blue box in Figure 8), and the 4th element is [MEM(1,4),MEM(2,2)];
- The dataset denoted by set64_128_256 is based on:
  Three levels of MEM denoted by 64, 128, and 256, where the size of segment equals min(64,128,256)=64, the number of segments equals $N_{EMr}/N_{ss}=32$, and a single element is a three-dimensional vector of features—e.g., the 3rd element is [MEM(1,3),MEM(2,2),MEM(3,1)] or the 7th element is [MEM(1,7),MEM(2,4),MEM(3,2)] (yellow box in Figure 8).

Each set was further utilized during the classification process independently, meaning that one classifier run was based on only one feature set. Such a set was divided into training and test sets, with the usage of the *leave-one-out cross-validation* approach. The following division rule was applied: in each classifier run, $N_{pp}$ entropy maps—calculated for one participant and for one session—were always left for defining a test set. The number of samples in the training and test sets ($N_{trs}$ and $N_{tss}$, respectively) may be expressed by means of formulas defined in Equations (6) and (7).

(6) $$N_{trs}=N_{pp}\times \left(N_{p}-1\right)\times size\left(setX\_Y\_Z\right)$$

(7) $$N_{tss}=N_{pp}\times size\left(setX\_Y\_Z\right)$$

where

(8) $$size\left(setX\_Y\_Z\right)=N_{ss}\times 2^{l_{X}-1}$$

where setX_Y_Z represents a set of feature vectors used to build the training and test sets, and $l_{X}$ denotes the level of the lowest segment size, which is used in calculating a feature, (for setX_Y_Z X=min(X,Y,Z) thus if set64_128_256 then X=64 so $l_{X}=1$).

There were $N_{ps}$ classifier runs for one *k* value and $7\times N_{ps}=322$, when all *k* values were taken into consideration. Depending on the segment used, there was a different number of classes ($N_{cl}$) in a dataset:

(9) $$N_{cl}\left(setX\_X\_Z\right)=\frac{N_{EMr}}{N_{ss}\times 2^{l_{X}-1}}$$

which gives:

- 32 for datasets denoted by set64, set64_128, set64_128_256, set64_128_256_512;
- 16 for set128, set128_256, set128_256_512;
- 8 for set256, set256_512;
- 4 for set512.

In each set, classes were well-balanced—they were represented by an equal number of samples, $N_{ps}\times N_{pp}$. The obtained results were averaged over all MS series and presented in Table 1.

It can be easily noted that the overall classification performance is unsatisfactory, even in the case of the outcome obtained for the segment size equal to 512 and *k* equal to 255, which, despite being the best, is still worse than a random draw.

This finding influenced the next step of the analysis. The research question was: Were all eye movement segments recognized with the same level of accuracy, or was a better outcome obtained for some of them? In order to answer this question, the performance of the prediction to which part of an eye movement signal a particular sample belongs to, was assessed. It was achieved by averaging classification results separately for each segment defined in a particular set. For example, in the case of set64, there were 32 segments: 1–64, 64–128, 128–256 …; thus, we obtained 32 classification results, averaged over all EM series. Subsequently, the segment with the best classification accuracy was selected. This procedure was repeated for all ten datasets and all *k*-values. All the chosen segments within each set, with the corresponding results, are shown in Table 2.

When studying the presented results (Table 2), it may be seen that the best and the worst classification performances were revealed for sets with the longest (set512) and the shortest (set64) eye-movement segments, respectively. However, good results were also obtained for sets built based on feature vectors consisting of more than one feature, such as set256_512 or set128_256_512. The outcomes calculated for parameters *k127* and *k255* are comparable with those estimated for set512 and *k3* or *k7*. Analysis of the second column in Table 2 points out that the best classification results were obtained for similar eye-movement segments, all belonging to the first 512 ms of eye movement after a stimulus position shift.

Exploration of the results for the remaining segments exposed the weaker efficiency of the classifier. Example outcomes obtained for one dataset and all classifier runs are shown in Table 3. This includes results for set256, notwithstanding how a similar classification pattern was uncovered for the other classified sets.

**Figure 9:**
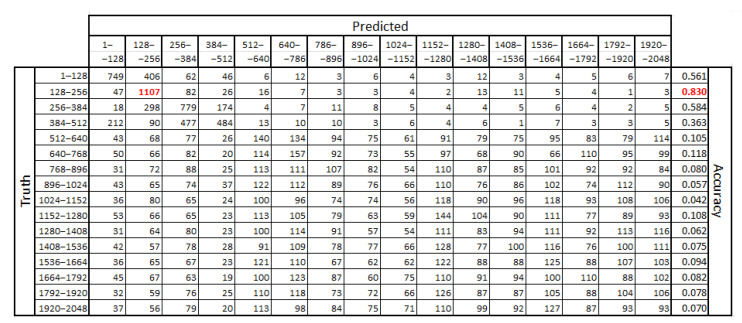
The example confusion matrix obtained for set128_256_512 with a k-value equal to 255. The best results were marked in red.

The contents of Table 3 disclose that the best performance in recognizing eye-movement scopes is in the first of the analyzed segments. It is much better than the values calculated for other segments, independent of the parameter *k*. A similar dependency was found in other datasets. However, depending on the segment size used, the best outcomes were revealed either in the second, third, or fourth segment. This is visible in a confusing matrix in Figure 9. Because it was prepared for set128_256_512, the segment size is equal to 128 and the best results were obtained for segment two: 128–256 (marked in red).

The sensitivity and specificity were calculated for the class with the best classification accuracy (128–256). They amounted to 0.83 and 0.921, respectively.

## 4. Discussion

The results presented in the previous section provided some premises which allowed treating the multilevel entropy map as a useful tool for describing hidden eye-movement dynamics.

### 4.1. The Multilevel Entropy Map

When analyzing entropy maps in Figure 5, quantitative differences and qualitative similarities can be perceived. The differences in entropy are visible between MEM A and MEM B, prepared for individual EM series, which confirms the idiosyncratic features of eye-movement dynamics. However, ranges of the variation coincide for both maps, as well as for the map of averaged entropy (MEM C). Based on these ranges and signal recordings, some conclusions regarding eye-movement events may be drawn. As mentioned earlier, by comparison of the courses of eye-movement velocity (see Figure 6), with values at various levels of entropy maps A and B, a matching of the saccadic period to a set of MEM segments may be performed. The averaged entropy map (MEM C) validates the inference that the presented individual patterns are good representatives of all studied series. In our opinion, such analysis is significantly facilitated by the application of the multilevel entropy map. Achieving such information based only on one level would be difficult. On the one hand, in smaller segment sizes, the entropy values turned out to be similar and repeatable. However, on the other hand, greater segments had precision too low to indicate the saccadic period accurately enough. By combining results from more than one level, more precise information became available. Nonetheless, because it can be imagined that visual exploration of the MEM for multiple data series may be unfeasible, a mechanism for automating detection had to be developed.

### 4.2. Eye Movement Events Detection

**Figure 10:**
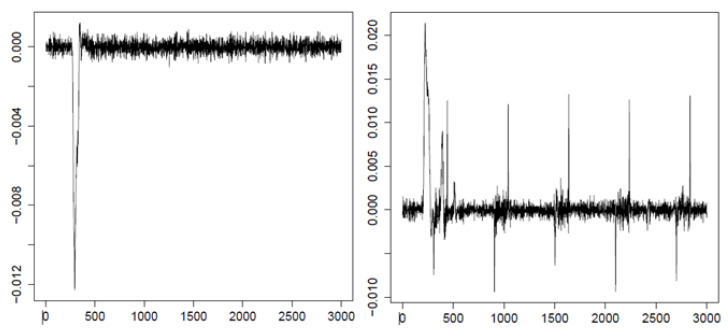
Two example courses of eye-movement velocity (the vertical axis). The horizontal axis represents time.

During assessment of the general classification outcomes, as illustrated in Table 1, the low classifier efficiency has to be noted. However, such results may be easily explained when looking at the example courses of eye movement velocity presented in Figure 10.

In the greater part of the signal, a similar characteristic may be perceived. Only at the beginning, when the saccade occurs, notable differences are visible. For this reason, entropy values describing more than half of the course (representing fixation) have a similar value in defined segments. This fact was also confirmed by statistical analysis, which exposed a lack of significant differences in the entropy evaluated for this part of the eye-movement velocity. This finding leads to the conclusion that for the purpose of classification improvement, shorter signal courses could be used. Such a solution is justified by the fact that fixations are usually shorter than 2000 ms. Therefore, the classification process was repeated only for the first half of the EM series. As expected, the classification performance increased; however, it regarded mostly those segments where fixations took place. For the results of the saccadic period, although they were improved as well, there were not any significant changes. The characteristic of this signal part was significantly different from the fixation scope; thus, shortening the latter had a small influence on the classification results for the former period.

The analysis of results in Table 2 showed that the worst accuracy was obtained for segments with the lowest size (set64). One of the reasons for such an outcome is the significantly different number of classes—compared to the best results (set512)—to which a particular sample had to be assigned. In the case of set512, it is one out of four classes, and in the case of set64, one out of 32. The second reason may be related to the fact that short parts of a signal are more similar to one another than longer ones, and it is more difficult to differentiate between them. Confirmation for this conclusion may be found in Figure 5, when studying the first levels of entropy maps. The values presented in the particular cells are very similar. The influence of a smaller-sized dataset—than that suggested for calculating approximate entropy— has to also be taken into account.

Contrary to set64, set512 yielded the best classification efficiency. However, despite providing the best classification accuracy, a segment size equal to 512 seems to be too wide to prove useful in reaching this study’s aims. Thus, attention should be focused on the sets with longer feature vectors, yet with shorter segments.

The second finding regarding this group of results is the influence of the feature vector length on the classification efficiency. It turned out that utilizing values from more than one level of the entropy map entailed better classification accuracy only for a specific group of eye-movement segments. For example, joining values from the lowest level (set64) with the arbitrary combination of values from the other levels brought about an expected improvement in the prediction (rows 1, 5–7 in Table 2). Similarly, by incorporating the feature vector values from the second level (set128) and from levels three and four (set256 and set512), the classifier yielded better results than when only values from the second level were applied (rows 2 and 9–10). However, when comparing rows 2 and 3 with row 8, a decrease in efficiency may be noted.

Collating the outcomes from this research with those obtained in [26], one may notice differences in the values of calculated entropy. This may stem from several reasons. The most important is the selection of values for the parameter *m* (in Equation (1)). The dimension of a hidden dynamical system can be estimated from the data by the use of the False Nearest Neighbors method [40], and may differ for different data series. Such a solution was utilized in [26]. In the current studies, one value, m=2 for all analyzed EM series was used, as recommended in [27] for approximate entropy usage. On the contrary, the sample entropy method was utilized in the above-mentioned research. Additionally, both studies explored slightly different sizes of eye-movement segments.

Comparing our method to ones previously described, we have to emphasize that it introduces a novel approach in the analyzed field. By the application of approximate entropy and MEM for determining the saccadic period, we avoided the necessity of any threshold usage. All required values were derived from raw data. Furthermore, in the classification task, the feature vectors, consisting only of two or three elements, were sufficient to achieve results at a very good level. Additionally, we also verified whether and how these outcomes depended on the dataset size. Thus, the previously used set of EM series was decreased by half. For division purposes, four rules were applied: (1) Only the EM series from the first session was taken into account; (2) only the EM series from the second session was utilized; (3) half of the EM time-series were taken from the first session and half from the second one, yet from other participants; (4) half of the EM time-series were taken from the first session and half from the second one, however, this time they were obtained randomly. The classification process was repeated for the set128_256_512, utilizing the same cross-validation type. The performance for *k* equaling to 255, identified as the best, was compared with the previously evaluated accuracy (0.830). For the first subset, the classifier efficiency was slightly lower at 0.786, while for the others, they were even slightly better and amounted to 0.834, 0.847, 0.853, respectively. This indicates that the method is able to yield good results even for shorter datasets, which may also be an advantage in regard to other methods.

Furthermore, approximate entropy was recognized by its ability to distinguish between different systems’ dynamics when short-length data with moderate noise are available [36]. For this reason, the proposed method may also prove useful for processing unfiltered data, as it was in the presented studies.

Despite providing promising results, the described solution has some limitations. The saccadic period able to be recognized by means of this method amounts to 128 ms, which, compared to the saccade duration (from 30 to 80 ms), is still too wide. For this reason, further improvements are required. We plan to utilize other approaches for evaluating entropy methods. The promising results in research regarding EEG signals with the application of fuzzy entropy and its multi-scale representation [41,42,43,44] encourage us to apply this method in the eye-movement research area, too.

Additionally, a fusion of entropy and chaotic signal description (such as the Largest Lyapunov Exponent) is planned to be verified in future investigations. Moreover, the proposed method will be applied in regard to both data series based on vertical eye-movement velocity and to other datasets. New experiments will be developed for this purpose, with the usage of other types of eye trackers. Various applications of the multilevel entropy map in other eye-movement-correlated domains supporting biometric identification or disease detection are also planned.

## 5. Conclusions

The aim of the presented research was to develop a new approach which may prove useful in describing eye-movement dynamics directed toward detection of eye-movement events. The multilevel entropy map seems to meet these requirements. We obtained satisfactory experimental results of the classification, which justify usage of the approximate entropy as a potential method for description of eye-movement dynamics. Nonetheless, as the performance of the presented method may be improved on, further studies are planned in this field.

## Figures and Tables

**Table 1:** The averaged results—the percentage of the correctly classified samples for various feature vector lengths and *k* values.

Set	k3	k7	k15	k31	k63	k127	k255
set64	0.034	0.034	0.034	0.033	0.035	0.033	0.037
set128	0.085	0.092	0.098	0.103	0.103	0.102	0.102
set256	0.206	0.217	0.226	0.231	0.229	0.229	0.233
set512	0.4201	0.437	0.444	0.458	0.453	0.456	0.466
set64_128	0.047	0.052	0.055	0.059	0.059	0.061	0.059
set128_256	0.138	0.148	0.153	0.157	0.157	0.159	0.161
set256_512	0.275	0.303	0.308	0.309	0.307	0.311	0.307
set64_128_256	0.072	0.079	0.084	0.087	0.087	0.087	0.087
set128_256_512	0.177	0.196	0.200	0.206	0.204	0.207	0.207
set64_128_256_512	0.090	0.102	0.106	0.109	0.112	0.111	0.107

**Table 2:** The best results—the percentage of correctly classified samples with corresponding segment sizes, averaged over all EM series.

Set	segment	k3	k7	k15	k31	k63	k127	k255
set64	128–192	0.053	0.071	0.079	0.076	0.086	0.121	0.186
set128	128–256	0.599	0.682	0.723	0.742	0.716	0.705	0.720
set256	1–256	0.618	0.677	0.730	0.749	0.718	0.703	0.721
set512	1–512	0.802	0.852	0.876	0.900	0.915	0.935	0.936
set64_128	192–256	0.202	0.258	0.317	0.361	0.392	0.439	0.462
set128_256	128–256	0.527	0.606	0.643	0.679	0.672	0.675	0.705
set256_512	1–256	0.670	0.7423	0.774	0.792	0.819	0.840	0.858
set64_128_256	128–192	0.283	0.324	0.349	0.355	0.373	0.401	0.430
set128_256_512	128–256	0.564	0.6664	0.699	0.739	0.775	0.809	0.830
set64_128_256_512	128–192	0.310	0.343	0.387	0.402	0.442	0.471	0.499

**Table 3:** The results—i.e., the percentage of correctly classified samples—obtained for a segment size equal to 256 (set256), averaged over all EM series.

Segment	k3	k7	k15	k31	k63	k127	k255
1–256	0.618	0.677	0.730	0.749	0.718	0.703	0.721
257–512	0.188	0.171	0.197	0.202	0.232	0.268	0.2945
513–768	0.156	0.164	0.158	0.174	0.202	0.178	0.185
769–1024	0.139	0.144	0.136	0.136	0.123	0.127	0.130
1025–1280	0.151	0.151	0.157	0.163	0.148	0.119	0.097
1291–1536	0.150	0.144	0.148	0.118	0.127	0.150	0.166
1537–1792	0.137	0.149	0.133	0.1567	0.160	0.169	0.122
1793–2048	0.147	0.171	0.142	0.146	0.118	0.096	0.140

## Supplementary Materials

Data set used in the research is available online at http://kharezlak.pl/eyetracking.html.

## Abbreviations

**Abbreviation**	**Description**	**Value**
$N_{pp}$	Number of point positions	29
$N_{p}$	Number of participants	24
$N_{spp}$	Number of sessions per participant	2
$N_{ps}$	Number of participant sessions	46
$N_{EMs}$	Number of eye movement series	1334
$N_{EMr}$	Number of eye movement recordings	2048
$N_{ss}$	Number of samples in the smallest segment of eye movement recordings	64

## References

[1] Poole A., Ball L.J. (2006). Eye tracking in HCI and usability research. Encyclopedia of Human Computer Interaction.

[2] Harezlak K., Rzeszutek J., Kasprowski P. (2015). The Eye Tracking Methods in User Interfaces Assessment. Intelligent Decision Technologies, Proceedings of the 7th KES International Conference on Intelligent Decision Technologies (KES-IDT 2015), Sorrento, Italy, 21–23 June 2017.

[3] Foster T.E., Ardoin S.P., Binder K.S. (2013). Underlying changes in repeated reading: An eye movement study. Sch. Psychol. Rev..

[4] Hyona J., Lorch R.F., Kaakinen J.K. (2002). Individual differences in reading to summarize expository text: Evidence from eye fixation patterns. J. Educ. Psychol..

[5] Jarodzka H., Scheiter K., Gerjets P., Van Gog T. (2010). In the eyes of the beholder: How experts and novices interpret dynamic stimuli. Learn. Instr..

[6] Harezlak K., Kasprowski P., Kasprowska S. (2017). Eye Movement Traits in Differentiating Experts and Laymen. Man-Machine Interactions 5. ICMMI 2017.

[7] Harezlak K., Kasprowski P. (2018). Application of eye tracking in medicine: A survey, research issues and challenges. Comput. Med. Imaging Graph..

[8] Mele M.L., Federici S. (2012). Gaze and eye-tracking solutions for psychological research. Cogn. Process..

[9] Palinko O., Kun A.L., Shyrokov A., Heeman P. Estimating cognitive load using remote eye tracking in a driving simulator. Proceedings of the 2010 Symposium on Eye-Tracking Research & Applications.

[10] Holmqvist K., Nyström M., Andersson R., Dewhurst R., Jarodzka H., Van de Weijer J. (2011). Eye Tracking: A Comprehensive Guide to Methods and Measures.

[11] Otero-Millan J., Troncoso X.G., Macknik S.L., Serrano-Pedraza I., Martinez-Conde S. (2008). Saccades and microsaccades during visual fixation, exploration, and search: Foundations for a common saccadic generator. J. Vis..

[12] Martinez-Conde S., Otero-Millan J., Macknik S.L. (2013). The impact of microsaccades on vision: Towards a unified theory of saccadic function. Nat. Rev. Neurosci..

[13] Darrien J.H., Herd K., Starling L.J., Rosenberg J.R., Morrison J.D. (2001). An analysis of the dependence of saccadic latency on target position and target characteristics in human subjects. BMC Neurosci..

[14] Zemblys R., Niehorster D.C., Komogortsev O., Holmqvist K. (2018). Using machine learning to detect events in eye-tracking data. Behav. Res. Methods.

[15] Salvucci D.D., Goldberg J.H. (2000). Identifying Fixations and Saccades in Eye-tracking Protocols. Proceedings of the 2000 Symposium on Eye Tracking Research & Applications (ETRA ’00).

[16] Nyström M., Holmqvist K. (2010). An adaptive algorithm for fixation, saccade, and glissade detection in eyetracking data. Behav. Res. Methods.

[17] Mould M.S., Foster D.H., Amano K., Oakley J.P. (2012). A simple nonparametric method for classifying eye fixations. Vis. Res..

[18] Larsson L., Nyström M., Stridh M. (2013). Detection of Saccades and Postsaccadic Oscillations in the Presence of Smooth Pursuit. IEEE Trans. Biomed. Eng..

[19] Larsson L., Nyström M., Andersson R., Stridh M. (2015). Detection of fixations and smooth pursuit movements in high-speed eye-tracking data. Biomed. Signal Process. Control.

[20] Hessels R.S., Niehorster D.C., Kemner C., Hooge I.T.C. (2017). Noise-robust fixation detection in eye movement data: Identification by two-means clustering (I2MC). Behav. Res. Methods.

[21] Astefanoaei C., Creanga D., Pretegiani E., Optican L., Rufa A. (2014). Dynamical Complexity Analysis of Saccadic Eye Movements In Two Different Psychological Conditions. Roman. Rep. Phys..

[22] Murata A., Matsuura T., Kurosu M. (2015). Nonlinear Dynamical Analysis of Eye Movement Characteristics Using Attractor Plot and First Lyapunov Exponent. Human-Computer Interaction: Interaction Technologies.

[23] Harezlak K. (2017). Eye movement dynamics during imposed fixations. Inf. Sci..

[24] Harezlak K., Kasprowski P., Czarnowski I., Howlett R.J., Jain L.C. (2018). Chaotic Nature of Eye Movement Signal. Intelligent Decision Technologies 2017.

[25] Rosenstein M.T., Collins J.J., De Luca C.J. (1993). A Practical Method for Calculating Largest Lyapunov Exponents from Small Data Sets. Phys. D Nonlinear Phenom..

[26] Harezlak K., Kasprowski P. (2018). Searching for Chaos Evidence in Eye Movement Signals. Entropy.

[27] Pincus S.M. (1991). Approximate entropy as a measure of system complexity. Proc. Natl. Acad. Sci. USA.

[28] Engoren M. (1998). Approximate entropy of respiratory rate and tidal volume during weaning from mechanical ventilation. Crit. Care Med..

[29] Bruhn J., Röpcke H., Hoeft A. (2000). Approximate entropy as an electroencephalographic measure of anesthetic drug effect during desflurane anesthesia. Anesthesiol. J. Am. Soc. Anesthesiol..

[30] Vukkadala S., Vijayalakshmi S., Vijayapriya S. (2009). Automated detection of epileptic EEG using approximate entropy in elman networks. Int. J. Recent Trends Eng..

[31] Karmakar C.K., Khandoker A.H., Begg R.K., Palaniswami M., Taylor S. (2007). Understanding Ageing Effects by Approximate Entropy Analysis of gait variability. Conf. Proc. IEEE Eng. Med. Biol. Soc..

[32] Castiglioni P., Rienzo M.D. How the threshold “r” influences approximate entropy analysis of heart-rate variability. Proceedings of the 2008 Computers in Cardiology.

[33] Li X., Jiang Y., Hong J., Dong Y., Yao L. (2016). Estimation of cognitive workload by approximate entropy of EEG. J. Mech. Med. Biol..

[34] Jazz-Novo (2018). Ober Consulting. http://www.ober-consulting.com/9/lang/1/.

[35] Pincus S.M., Huang W.M. (1992). Approximate entropy: Statistical properties and applications. Commun. Stat. Theory Methods.

[36] Pincus S. (1995). Approximate entropy (ApEn) as a complexity measure. Chaos Interdiscip. J. Nonlinear Sci..

[37] Yentes J., Hunt N., Schmid K., Kaipust J., McGrath D., Stergiou N. (2013). The appropriate use of approximate entropy and sample entropy with short data sets. Ann. Biomed. Eng..

[38] Borchers H.W. (2018). Package ‘Pracma’. https://cran.r-project.org/web/packages/pracma/pracma.pdf.

[39] Kasprowski P., Harezlak K., Czarnowski I., Caballero A.M., Howlett R.J., Jain L.C. (2016). Using Dissimilarity Matrix for Eye Movement Biometrics with a Jumping Point Experiment. Intelligent Decision Technologies 2016: Proceedings of the 8th KES International Conference on Intelligent Decision Technologies (KES-IDT 2016)—Part II, Tenerife, Spain 15–17 June 2016.

[40] Kennel M.B., Brown R., Abarbanel H.D.I. (1992). Determining embedding dimension for phase-space reconstruction using a geometrical construction. Phys. Rev. A.

[41] Cao Z., Lin C.T. (2018). Inherent fuzzy entropy for the improvement of EEG complexity evaluation. IEEE Trans. Fuzzy Syst..

[42] Azami H., Fernández A., Escudero J. (2017). Refined multiscale fuzzy entropy based on standard deviation for biomedical signal analysis. Med. Biol. Eng. Comput..

[43] Liu C., Li K., Zhao L., Liu F., Zheng D., Liu C., Liu S. (2013). Analysis of heart rate variability using fuzzy measure entropy. Comput. Biol. Med..

[44] Liang Z., Wang Y., Sun X., Li D., Voss L.J., Sleigh J.W., Hagihira S., Li X. (2015). EEG entropy measures in anesthesia. Front. Comput. Neurosci..

